# Trajectories and influencing factors of psychological distress in nasopharyngeal carcinoma patients receiving radiotherapy (incorporating genetic factors): a multicenter longitudinal study

**DOI:** 10.3389/fonc.2025.1640266

**Published:** 2025-08-27

**Authors:** Jiajie Ning, Jianhua Huang, Yan Liao, Jianxiong Long, Pingping Zeng

**Affiliations:** ^1^ Nursing College, Guangxi Medical University, Nanning, China; ^2^ Department of Epidemiology and Health Statistics, School of Public Health of Guangxi Medical University, Nanning, China

**Keywords:** nasopharyngeal carcinoma, latent class growth model, distress, circRNA, CircRNA-RBP network, circRNA-miRNA-mRNA network

## Abstract

**Background:**

This multicenter longitudinal study seeks to investigate the dynamic changes of psychological distress (PD) in nasopharyngeal carcinoma (NPC) patients during radiotherapy, and reveal the expression profiles and regulatory networks of circRNAs in these NPC patients.

**Methods:**

282 newly diagnosed NPC patients from three hospitals in China were included. Participants completed questionnaires and provided blood samples. PD trajectories were identified via a latent class growth model (LCGM). Moreover, the factors that influence the PD trajectories were explored. Whole transcriptome sequencing was performed to investigate genetic factors. The real-time quantitative PCR was applied to validate circRNAs. We predicted the target miRNAs, target mRNAs and target RNA-binding proteins (RBPs) of the top 10 malregulated circRNAs. Subsequently, circRNA-miRNA-mRNA (ceRNA) and circRNA-RBP networks were constructed. In addition, the role of circRNA and target mRNA parent genes was predicted by KEGG and GO analysis.

**Results:**

LCGM identified two of the most important PD trajectories during radiotherapy in NPC patients: Class 1 “decline distress group” (11.0%) and Class 4 “rise distress group” (20.3%). Household monthly per capita income, coping strategies, and perceived social support emerged as important predictors of PD trajectories. Regarding genetic factors, 600 circRNAs and 123 miRNAs were identified as being significantly differentially expressed. Notably, hsa_circ_0004277 demonstrated significant differences between patients in the rise and decline distress groups (P < 0.01). ceRNA and RBP networks may influence the pathophysiology of PD in NPC patients undergoing radiotherapy.

**Conclusion:**

This study unraveled that PD trajectories in NPC patients during radiotherapy were heterogeneous, indicating the need for screening and timely interventions within this population. Furthermore, the expression patterns of ceRNA and circRNA–RBP networks and pathways related to these networks suggested a potential role of circRNAs in developing PD among NPC patients receiving radiotherapy.

## Background

Nasopharyngeal carcinoma (NPC) represents a type of cancer that originates in the nasopharyngeal mucosa. New cases of NPC worldwide exceeded 120416 in 2022, with over 73476 deaths, according to the International Agency for Research on Cancer. Moreover, males have notably higher incidence and death rates of NPC compared to females (1). In China, the age-standardized mortality rate of NPC reduced from 2.0 per 100000 in 2005 to 1.4 per 100000 in 2020, with an annual average percent change of -2.4%. A declining trend was also observed in years of life loss (2). Currently, radiotherapy, which effectively controls tumor progression and prolongs survival, serves as the primary treatment for NPC. Recent reports from NPC treatment centers have revealed encouraging clinical outcomes. Four large-sample studies (3–6) reported 5-year overall survival rates above 80% and 5-year local control rates ranging from 88% to 95%. Nevertheless, previous studies (7, 8) have demonstrated that following radiotherapy, NPC patients often experience significant psychological distress (PD), which may be caused by adverse effects including dry mouth (9), vomiting (10), taste loss (11), and appetite loss (12). PD represents an unpleasant experience of psychosocial (cognitive, behavioral, and emotional) alterations caused by multiple factors according to the National Comprehensive Cancer Network (NCCN). This poor emotional experience substantially impacts a patient’s life and ability to cope with NPC, thereby reducing treatment efficacy (13). A study has suggested that factors including personality type, coping strategies, and perceived social support (PSS) are closely linked to PD development in cancer patients (14, 15).

Most cross-sectional studies to date have revealed a high prevalence of PD among NPC patients undergoing radiotherapy. Buchmann et al. (16) found that patients with head and neck cancers, including NPC, had distress scores approaching moderate levels after radiotherapy. Another study (17) unraveled that nearly one-third of NPC patients had distress scores exceeding the critical threshold, with distress levels notably increasing during concurrent chemoradiotherapy. However, current studies on PD among NPC patients receiving radiotherapy are limited to cross-sectional designs, which do not capture the evolving psychological states of patients throughout the treatment period. Therefore, this longitudinal multicenter study focused on investigating the dynamic trajectories of PD in NPC patients undergoing radiotherapy and identifying the factors influencing these trajectories. Their trajectories were identified via the latent class growth model (LCGM) (18). LCGM stands as a statistical method designed for longitudinal studies. LCGM can identify unobserved subgroups within a population based on their longitudinal data. LCGM is particularly valuable for studying PD in cancer patients undergoing radiotherapy, as it classifies patients into distinct trajectories based on their dynamic changes. Previous studies have utilized LCGM to investigate PD trajectories and influencing factors in prostate cancer patients undergoing radiotherapy (19) and provided personalized interventions for different categories of patients. Moreover, another study (15) identified three distinct PD trajectories in gynecologic cancer patients and explored potential intervention strategies. Thus, identifying high levels of PD in cancer patients for timely, personalized interventions is crucial for promoting their psychological recovery.

Emerging evidence has unraveled a potential correlation between genetic factors and PD among cancer patients receiving radiotherapy. CircRNAs are single-stranded, endogenous, and non-coding RNAs discovered over the past two decades. CircRNAs have been shown through high-throughput sequencing to play crucial roles in various biological functions (20). CircRNAs regulate the expression patterns of target mRNAs by binding to miRNAs, modulating gene expression during various cellular processes, translating into proteins, as well as interacting with RNA-binding proteins (RBPs) (21). Some studies have linked circRNAs to an increased risk of psychological disorders. Nevertheless, studies on circRNAs in cancer patients with PD are scarce. One study (22) built a circRNA–miRNA–mRNA network for depression and cancer. It identified 28 notably upregulated circRNAs, such as hsa_circ_0007334, highlighting their potential prognostic and diagnostic value in clinical practice. Hence, it is plausible to hypothesize that circRNAs might impact PD in NPC patients undergoing radiotherapy, potentially serving as diagnostic and therapeutic biomarkers. Unfortunately, no study validated this hypothesis in NPC patients receiving radiotherapy.

This multicenter cohort study analyzed multiple measurements of PD (recorded at four time points: admission, after the first radiotherapy session, after the 15th session, and after the 30th session) in NPC patients undergoing radiotherapy to describe the distress changes throughout the treatment process. This study explored the influencing factors of PD, including coping strategies, personality type, and PSS, combined with genetic analysis. A ceRNA network and circRNA–RBP networks were built via whole-transcriptome sequencing (WTS) to identify potential risk genes linked to PD in NPC patients undergoing radiotherapy. Factors influencing PD trajectories in these NPC patients were investigated by analyzing the target genes obtained from the ceRNA network and the maternal genes of circRNAs through KEGG and GO analyses. This study aims to provide scientific references for alleviating both physical discomfort and negative emotions in NPC patients undergoing radiotherapy.

## Methods

### Participants and ethics statement

This multicenter longitudinal study was conducted at three tertiary Grade A hospitals (A, B, and C) located in an NPC endemic area in China. All participants were informed by a doctor or nurse about the purpose and significance of this study, and their rights and obligations, and they all provided informed consent. Patients were enrolled consecutively upon admission between July 2024 and February 2025. Baseline data and an electronic mental health survey were collected during their hospitalization (The patient selects the paper or electronic questionnaire to fill in the questionnaire). Participants completed a questionnaire at each time point and provided blood samples for immune evaluation at admission and after the 30th radiotherapy session.

The inclusion criteria were: i) age ≥18 years; ii) pathological diagnosis of WHO type II-IV NPC within 1 week; iii) having stage I-IVB disease [UICC/AJCC Classification (8th edition)]; iv) NPC patients first receiving radiotherapy (intensity-modulated radiation therapy) with chemotherapy and complete it within 90 days; v) sufficient cognitive and language abilities to finish the electronic questionnaire independently. The exclusion criteria were: i) participating or participated in psychological intervention experimental studies; ii) having heart, brain, lung, liver, kidney, or other organ diseases. This study was carried out following the Declaration of Helsinki and approved by the Ethics Review Committee of Guangxi Medical University (Project No. KY0172, 2024).

### Sample size estimation

The sample size of the longitudinal study was calculated by GLIMMPSE software. Hotelling Lawley Trace (23) was selected as the statistical test for the LCGM, with a significance level of 0.05 and statistical power of 0.90 (β = 0.10). Based on preliminary investigation in this study, NPC patients typically undergo 30 to 33 radiotherapy sessions over a period of 45 to 60 days. As radiotherapy-related adverse effects may accumulate over time, the total number of sessions was evenly divided to assess patients’ PD at different stages, namely at baseline (before treatment), after 15 sessions (mid-treatment), and after 30 sessions (post-treatment).

Repeated measures were specified and four time points were established: at admission (T1), after the first radiotherapy session (T2), after the 15th session (T3), and after the 30th session (T4). The estimated sample size was 190. Considering the potential for attrition or dropout in longitudinal studies, a 20% attrition rate was allowed in this study. Therefore, the required sample size increased to 190/(100%-20%) =238. Therefore, at least 238 NPC patients undergoing radiotherapy should be enrolled in this study. A sampling ratio of 3:1:1 was determined based on the annual admission numbers for NPC patients receiving radiotherapy at the three hospitals. At least, it was expected to collect 136, 46, and 46 cases in hospitals A, B, and C respectively.

### Survey instruments

#### Psychological distress

PD was evaluated via the Distress Thermometer (DT) (24). A 0–10 numerical rating scale was applied, where 0 indicated no distress and 10 represented extreme distress. A DT score ≥4 suggested clinically significant distress, and a score ≥7 indicated severe distress according to the NCCN.

#### Medical Coping Modes Questionnaire

Individuals’ coping strategies, encompassing surrender, confrontation, and avoidance, were assessed by MCMQ (25). Patients completed the questionnaire by selecting one of four response options for each item scored on a 1–4 scale. Higher scores on a particular coping strategy indicated a greater propensity to use that strategy.

#### Ten Item Personality Inventory

The TIP (26) is a validated, brief measure of the big five personality traits. This measure comprises five dimensions: openness, conscientiousness, emotional stability, extraversion, and agreeableness. 10 items scored on a 1–7 scale were assessed, with 1 suggesting strongly disagree and 7 indicating strongly agree. Higher scores on a particular dimension indicated more pronounced the corresponding personality trait.

#### Perceived Social Support Scale

The PSSS (27), developed by Dahlem and Zimet, comprises 12 items rated on a 1–7 scale. It assessed family support (four items, maximum score 28), friend support (4 items, maximum score 28), and other support (four items, maximum score 28). The total score was 84, with higher scores indicating higher PSS.

#### Total RNA extraction and the construction and sequencing of cDNA library

Peripheral blood (5 mL) was collected from participants who provided informed consent at T1 and T4. Using the PAXgene Blood RNA Kit (BD, USA), the total RNAs were extracted from samples of the rise distress group (Class 4) and decline distress group (Class 1) divided by LCGM following the manufacturer’s instructions. A Nanodrop ND-1000 (Thermo Fisher, USA) was applied to measure the concentration and quality of total RNAs. The sequencing library was built using around 1–2 μg of total RNAs per sample. RNA sequencing services were provided by CloudSeq Inc. (Shanghai, China). Trizol (Invitrogen, USA) was utilized to extract Total RNA, and rRNA was removed via the GenSeq^®^ rRNA Removal Kit (GenSeq, Inc., Shanghai, China). Directional RNA libraries were built via the GenSeq^®^ Directional RNA Library Prep Kit. RNA was fragmented to ~300 nt in length. Reverse transcriptase and random hexamers were utilized to synthesize the first-strand cDNA. Moreover, the dUTP Mix was applied to synthesize the second-strand cDNA. The double-stranded cDNA fragments underwent end repair, adapter ligation, and dA-tailing. The cDNA, ligated with adapters, was PCR-amplified and purified to generate the final sequencing libraries. Furthermore, the libraries were sequenced in paired-end 150 bp mode.

### Data processing

Paired-end reads were obtained through sequencing, and quality control was conducted via Q30. After quality control, high-quality reads were identified by filtering raw data with fastp software to remove adapters and low-quality reads. Pruned data were mapped to the reference genome. Expression profiles, differentially expressed transcripts, and differentially expressed genes (DEGs) were calculated. Volcano plots were generated to visualize DEGs. All data processing was carried out by CloudSeq Inc., and results were verified by two researchers to ensure accuracy for subsequent analysis.

### Construction of the circRNA-miRNA-mRNA network

Regarding the top 10 differentially expressed circRNAs (DEcircRNAs), their target miRNAs were predicted via RNAhybrid, circBank, CircInteractome, and TargetScan. Overlapping miRNAs were identified by intersecting the four databases and the differentially expressed miRNAs (DEmiRNAs). Target mRNAs for the intersecting miRNAs were predicted via MiRTarBase. A circRNA–miRNA–mRNA (ceRNA) network was built based on the intersecting miRNAs, the top 10 dysregulated DEcircRNAs, and the predicted mRNAs. The ceRNA network was visualized through Cytoscape 3.9.1. **Supplementary Table 1** illustrates the top 10 upregulated and downregulated circRNAs.

### Real-time quantitative PCR validation

The random three upregulated circRNAs identified in the ceRNA network were validated via RT-qPCR. Peripheral blood mononuclear cells were isolated from blood samples collected from Class 4 and Class 1 patients at T1 and T4 to extract total RNAs via Trizol (Invitrogen, USA). Gel electrophoresis and Spark 10 M (Tecan, Switzerland) were utilized to appraise RNA quality. Based on the manufacturer’s instructions, reverse transcription was carried out via PrimeScript™ RT Master Mix Kit (Takara, Japan). TB Green^®^ Premix Ex Taq™ (Takara, Japan) was utilized to amplify cDNA on the ABI StepOnePlus system (Applied Biosystems, USA). CircRNA primers were formed through backsplicing on the CircInteractome and National Center for Biotechnology Information website and were then synthesized by Sangon Biotech (Shanghai, China). The 2^−ΔCT method was utilized to calculate the data, with GAPDH was used as internal reference. Every group consisted of three technical replicates. The specificity of primers was verified by qRT-PCR experiments, and the results showed that the primer amplification efficiency was good and the specificity was strong. **Supplementary Table 2** lists all primer sequences.

### GO and KEGG analyses

The biological processes, cellular components, and molecular functions of genes can be explored through GO analysis. KEGG analysis reveals enriched pathways related to human diseases, metabolism, and cellular processes. Therefore, KEGG analysis can support constructing a regulatory network for exploring the development and progression of a disease. GO and KEGG analyses were carried out to reveal the impacts of circRNAs and miRNAs on the rise and decline distress group. The top 10 pathways based on the Enrichment Score (ES) were selected to explore the pathways related to the maternal genes of circRNAs and identified mRNAs in the ceRNA network. R version 4.4.1 was applied to conduct KEGG and GO analyses.

### Protein–protein interaction networks

Hub genes in NPC patients with PD were identified to further examine the ceRNA network. The STRING database (https://string-db.org/) was utilized to build a PPI network. Moreover, the PPI network was visualized by the CytoHubba plugin in Cytoscape (version 3.9.1) to obtain the top 10 genes within the ceRNA network. The PPI network included interactions with a combined score greater than 0.7.

### circRNA–RBP networks

Target RBPs for the top 10 dysregulated DEcircRNAs were predicted via circAtlas (28) and RBPsuite databases. The intersecting RBPs were screened by Venny 2.0. Furthermore, the predicted RBPs and dysregulated circRNAs were utilized to build a circRNA–RBP network. Cytoscape 3.9.1 was then applied to visualize the network.

### Statistical analysis

R version 4.4.1 was applied to carry out statistical analysis. Categorical data were presented as absolute counts and percentages, while continuous data were expressed as mean ± standard deviation for normally distributed variables, and median with interquartile ranges for non-normally distributed variables. Chi-square tests were applied to compare categorical data, and independent-sample t-tests were employed to compare normally distributed continuous data between two groups. Analysis of variance was used for multiple group comparisons, while the LSD-t test was applied to pairwise comparisons. Non-normally distributed data between two groups were compared via the non-parametric Wilcoxon test, while those among multiple groups were compared through the Kruskal–Wallis H test.

LCGM was analyzed via Mplus version 8.3. The model was tested with 1 to 5 classes sequentially. The average posterior probability were calculated for each participant in the model. This value represented the posterior probability of each individual being assigned to the corresponding trajectory group. An average posterior probability greater than 70% was considered acceptable. Model fit was evaluated by the sample size-adjusted BIC (aBIC), Bayesian Information Criterion (BIC), and Akaike Information Criterion (AIC). Smaller values indicated a better fit. The bootstrapped likelihood ratio test (BLRT) and Lo-Mendell-Rubin likelihood ratio test (LMR-LRT) were applied to compare k-class models with k−1 class models, with P-values < 0.05 indicating that the k-class model performed notably better. Entropy suggested classification accuracy, with values > 0.8 representing approximately 90% accuracy. A minimum proportion of 5% per class was required. Finally, logistic regression was carried out to explore factors influencing PD in NPC patients. Sociodemographic variables with statistical significance were included as independent variables, and latent classes of PD as dependent variables. A two-sided significance level of α = 0.05 was applied.

## Results

### Study population

300 eligible patients were initially enrolled. During follow-up, nine patients were lost at T2, four at T3, and five at T4. Ultimately, 282 patients were enrolled in the study (170 from Hospital A, 56 from Hospital B, and 56 from Hospital C). **Table 1** illustrates the sociodemographic and clinical features of this population.

**Table 1:** Sociodemographic and clinical characteristics of NPC patients receiving radiotherapy.

Characteristic	Value
Age, mean ± SD (range) years	49.03 ± 11.27 (18-79)
Gender n (%)	
Male	194 (68.79%)
Female	88 (31.21%)
Occupational status n (%)	
Employed	215 (76.24%)
Retired	17 (6.03%)
Unemployed	50 (17.73%)
Education background n (%)	
Junior high school or below	166 (58.87%)
High school/vocational secondary school	69 (24.47%)
Junior college	30 (10.63%)
Bachelor’s degree or above	17 (6.03%)
Marital status n (%)	
Unmarried	29 (10.28%)
Married	238 (84.40%)
Divorced	9 (3.19%)
Widowed	6 (2.13%)
Smoking and drinking history n (%)	
Smoking	71 (25.18%)
Drinking	35 (12.41%)
Both smoking and drinking	115 (40.78%)
None	61 (21.63%)
Place of residence n (%)	
Urban	109 (38.65%)
Town	56 (19.86%)
Rural	117 (41.49%)
Healthcare payment method n (%)	
Urban resident medical insurance	69 (24.47%)
Employee medical insurance	59 (20.92%)
New rural cooperative medical system	154 (54.61%)
Household monthly per capita income n (%)	
≤1000 Yuan	72 (25.53%)
1001–3000 Yuan	97 (34.40%)
3001–5000 Yuan	96 (34.04%)
≥5001 Yuan	17 (6.03%)
Site of NPC	
Left side	135 (47.87%)
Right side	98 (34.75%)
Both	49 (17.38%)
Disease Stage n (%)	
II	24 (8.51%)
III	105 (37.23%)
IV	153 (54.26%)
Comorbidities	
Yes	124 (43.97%)
No	158 (56.03%)

### PD trajectories of NPC patients receiving radiotherapy

**Figure 1:**
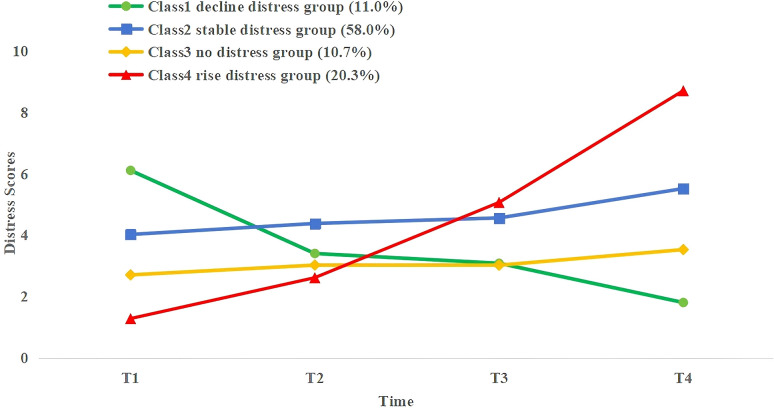
PD trajectories of NPC patients receiving radiotherapy.

LCGM on 282 NPC patients undergoing radiotherapy at T1, T2, T3, and T4 was built to identify the trajectories of PD. The fit results for the heterogeneous trajectories of PD are shown in **Table 2**. The five-class model did not meet the criteria (P > 0.05 in the likelihood ratio test). The two-, three-, and four-class models fit well as they all had entropy values >0.8, and the LMR-LRT and BLRT reached a significant level (P < 0.05) with each class representing more than 5% of the total. The four-class model had the lowest AIC(4232.533), BIC(4287.108), and aBIC values(4239.544), suggesting that it was the best-fitting model. In this model, 32 patients (11.0%) were assigned to Class 1, 163 (58.0%) to Class 2, 30 (10.7%) to Class 3, and 57 (20.3%) to Class 4. The PD trajectories of these four classes are illustrated in **Figure 1**.

**Table 2:** Proportion of patient assigned to each class.

Category	AIC	BIC	aBIC	Entropy	VLMR *P*	BLRT *P*	Poster probability(%)	Class probability(%)
1	4410.043	4431.873	4412.848	–	–	–	–	–
2	4313.331	4346.076	4317.537	0.852	0.006	<0.001	0.94/0.94	0.189/0.811
3	4279.361	4323.021	4284.969	0.831	0.002	<0.001	0.90/0.89/0.91	0.125/0.698/0.177
4	4232.533	4287.108	4239.544	0.903	<0.001	<0.001	0.88/0.86/0.84/0.84	0.110/0.580/0.107/0.203
5	4228.118	4293.608	4236.531	0.752	0.271	0.056	0.82/0.81/0.82/0.83/0.84	0.068/0.181/0.149/0.107/0.495

Each class was designated according to its trajectory. The DT cutoff score for PD was based on the NCCN recommendations (DT score ≥4). Class 1 (C1, green) patients exhibited relatively high PD levels at T1 and then showed an accelerated decline. Thus, C1 was defined as a decline distress group; Class 2 (C2, blue) patients showed low PD levels at T1 and remained stable across T2–T4. Thus, C2 was defined as a stable distress group; Class 3 (C3, yellow) patients had low PD levels at T1 and remained consistent with DT scores below 4 at T2–T4. Thus, C3 was defined as a no distress group; Class 4 (C4, red) patients exhibited the lowest PD levels at T1 but experienced a sharp increase during T2–T4, peaking at T4. Thus, C4 was defined as a rise distress group.

### Univariable analysis of influencing factors in rise, stable and decline distress groups

Among the four trajectories identified through LCGM, we found that when four types of trajectories were selected for single-factor analysis, the only meaningful influencing factor obtained was the PSS score, which would affect the subsequent analysis of influencing factors. Therefore, in order to eliminate potential selection biases, we consulted relevant experts and ultimately the rise, stable and decline distress groups were selected for univariable and multivariable logistic regression analyses to explore potential factors influencing PD trajectories. Univariable analysis (**Table 3**) indicated that household monthly per capita income, the coping and yielding strategies in medical coping modes, and PSS were significantly associated with the differences among the three PD trajectories in NPC patients undergoing radiotherapy (P < 0.05).

**Table 3:** Univariate analysis of influencing factors in rise, stable and decline distress groups.

Item		C1 (n=32)	C2 (n=163)	C4 (n=57)	*t/X^2^*	*P*
Age *(mean ± SD)*		51.81 ± 10.17	48.29 ± 10.79	51.47 ± 11.23	8.046	0.429
Gender	Male	17(53.13)	110(67.48)	39(68.42)	3.575	0.167
	Female	15(46.87)	53(32.52)	18(31.58)		
Occupational status	Employed	24(75.00)	140(85.89)	32(56.14)	9.559	0.147
	Retired	2(6.25)	3(1.84)	10(17.54)		
	Unemployed	6(18.75)	20(12.27)	15(26.32)		
Education level	Junior high school or below	18(56.25)	123(75.46)	32(56.14)	8.046	0.429
	High school/technical secondary school	3(9.38)	27(16.56)	15(26.32)		
	Junior college	1(3.12)	10(6.13)	9(15.79)		
	Bachelor’s degree or above	10(31.25)	3(1.75)	3(1.75)		
Marital status	Unmarried	2(6.25)	15(9.20)	5(8.77)	10.029	0.123
	Married	26(81.25)	144(88.44)	45(78.95)		
	Divorced	3(9.38)	1(0.61)	5(8.77)		
	Widowed	1(3.12)	3(1.75)	2(3.51)		
Smoking and drinking history	Smoking	8(25.00)	38(23.31)	12(21.05)	0.479	0.924
	Drinking	5(15.63)	15(9.20)	7(12.28)		
	Both smoking and drinking	14(43.74)	89(54.60)	28(49.12)		
	None	5(15.63)	21(22.89)	10(17.55)		
Place of residence	Urban	12(37.50)	80(49.08)	12(21.05)	1.922	0.382
	Town	10(31.25)	21(22.89)	15(26.32)		
	Rural	10(31.25)	62(28.03)	30(52.63)		
Payment method for healthcare	Urban residents’ medical insurance	10(31.25)	38(23.31)	15(26.32)	7.746	0.101
	Employees’ medical insurance	4(12.50)	21(22.89)	16(28.07)		
	New rural cooperative medical system	18(56.25)	104(53.80)	26(45.61)		
Household monthly per capita income	≤1000 Yuan	8(25.00)	21(22.89)	37(64.91)	23.171	<0.001
	1001–3000 Yuan	3(9.38)	85(42.14)	12(21.05)		
	3001–5000 Yuan	10(31.25)	53(32.52)	7(12.28)		
	≥5001 Yuan	11(34.37)	4(2.45)	1(1.76)		
Site of NPC	Left side	16(50.00)	82(50.31)	31(54.39)	4.744	0.577
	Right side	10(31.25)	53(32.52)	17(29.82)		
	Both	6(18.75)	28(17.17)	9(15.79)		
Disease Stage	II	2(6.25)	14(8.59)	6(10.52)	2.615	0.735
	III	13(40.63)	60(36.81)	20(35.09)		
	IV	17(53.12)	89(54.60)	31(54.39)		
Comorbidities	Yes	20(62.50)	53(32.52)	25(43.86)	1.152	0.142
	No	12(37.50)	110(67.48)	32(56.14)		
Distress scores *(mean ± SD)*		3.57 ± 0.95	4.05 ± 1.27	4.31 ± 1.23	2.333	0.331
MCMQ *(mean ± SD)*	Confrontation	19.81 ± 4.46	9.05 ± 3.27	6.96 ± 2.96	3.603	0.042
	Avoidance	16.77 ± 2.09	12.72 ± 2.55	12.49 ± 2.71	2.870	0.065
	Surrender	10.39 ± 3.46	15.63 ± 5.90	16.07 ± 7.02	3.045	0.038
PSSS *(mean ± SD)*		69.31 ± 6.89	49.80 ± 5.25	30.42 ± 4.98	18.704	0.011

### Logistic regression analysis of influencing factors in rise, stable and decline distress groups

A binary logistic regression analysis was performed using the rise, stable, and decline distress groups as dependent variables, while household income, coping and yielding strategies, and PSS were considered as independent variables (**Table 4**). Firstly, to comprehensively compare different PD trajectories, the rise distress group was used as the reference group. The results showed that, compared to the stable distress group, patients with a monthly income ≤ 1000 were more likely to be assigned to the rise distress group (OR = 0.448). Patients with higher coping strategy scores had a greater likelihood of being assigned to the stable distress group (OR = 1.535). Compared with the decline distress group, patients with a monthly income ≤ 1000 (OR = 0.316), a monthly income of 1001-3000 (OR = 0.275), and a higher score for yielding strategies (OR = 0.316) were more likely to be categorized into the rise distress group. Patients with higher coping strategy scores (OR = 1.825) and increased PSS (OR = 1.530) had a greater likelihood of being classified into the decline distress group. Furthermore, when the stable distress group was used as the reference, the results indicated that higher coping strategy scores (OR = 1.734) and increased PSS (OR = 1.108) were associated with a greater likelihood of being categorized in the decline distress group.

**Table 4:** Logistic regression analysis of influencing factors in rise, stable and decline distress groups.

Predictors	Rise vs. stable distress groups			Rise vs. decline distress groups			stable vs. decline distress groups		
	*P*	*OR*	*95%CI*	*P*	*OR*	*95%CI*	*P*	*OR*	*95%CI*
household income ≥5001(Ref)									
≤1000	0.007	0.448	0.250-0.801	0.040	0.316	0.105-0.951	0.074	2.783	0.907-8.540
1001-3000	0.776	0.859	0.301-2.452	0.020	0.275	0.175-0.432	0.472	0.786	0.408-1.515
3001-5000	0.512	0.809	0.429-1.525	0.673	1.169	0.566-2.414	0.809	1.087	0.551-2.147
Confrontation	0.005	1.535	1.141-2.062	0.004	1.825	1.212-2.749	0.001	1.734	1.275-2.395
Surrender	0.662	0.870	0.0464-1.628	0.040	0.316	0.105-0.951	0.472	0.786	0.408-1.515
PSSS	0.908	1.054	0.432∼2.572	0.021	1.530	1.313-1.784	0.001	1.108	1.070-1.148

### Expression profiles of circRNAs in rise and decline distress groups

After identifying four trajectories of PD, the genetic factors influencing the upward or downward PD changes during the radiotherapy process in NPC patients were further investigated. Therefore, three patients from Class 1 (decline distress group) and three patients from Class 4 (rise distress group) were randomly selected. WTS was then conducted based on the 12 blood samples collected at T1 and T4. 34444 circRNAs were obtained by intersecting the sequencing data collected from the Class 1 and Class 4 patients at T1 and T4. DEcircRNAs were identified based on the criteria as follows: adjusted p ≤ 0.05, log (FC) ≥ 1.0, and average counts per million ≥ 100. Finally, 600 DEcircRNAs were identified, with 189 being upregulated and 411 downregulated between the two groups (**Supplementary Figure 1**). As illustrated in **Supplementary Figure 1**, the expression patterns of circRNA between Class 4 patients and Class 1 patients could be clearly distinguished.

### Expression profiles of miRNAs for rise and decline distress groups

1257 miRNAs were identified by intersecting miRNA-sequencing data collected from three Class 1 and three Class 4 patients at T1 and T4. The screening criteria were as follows: log (FC) ≥ 1.0 and adjusted p ≤ 0.05. 123 DEmiRNAs were selected, with 61 significantly upregulated and 62 significantly downregulated between the two groups. Hierarchical clustering demonstrated that the miRNA expression profiles could be effectively distinguished between Class 4 and Class 1 patients (**Supplementary Figure 2**).

### Analysis of the maternal gene of circRNA

The upregulated and downregulated genes were used to carry out KEGG and GO analyses to investigate the functions of the maternal genes of the DEcircRNAs in NPC patients with PD. KEGG analysis unraveled that the upregulated genes were enriched in the polycomb repressive complex, neurotrophin signaling pathway, and MAPK signaling pathway. GO analysis demonstrated that the upregulated genes were enriched in positive regulation of inflammasome-mediated signaling pathways, chromosome segregation, and positive regulation of NLRP3 inflammasome complex assembly. Additionally, KEGG analysis demonstrated that the downregulated genes were enriched in the viral life cycle, RNA degradation, and the polycomb repressive complex. While GO analysis indicated that the downregulated genes were enriched in lymphocyte differentiation, mononuclear cell differentiation, and positive regulation of post-translational protein modification (**Supplementary Figure 3**).

### Construction of the circRNA–miRNA–mRNA network for PD in NPC patients

**Figure 2:**
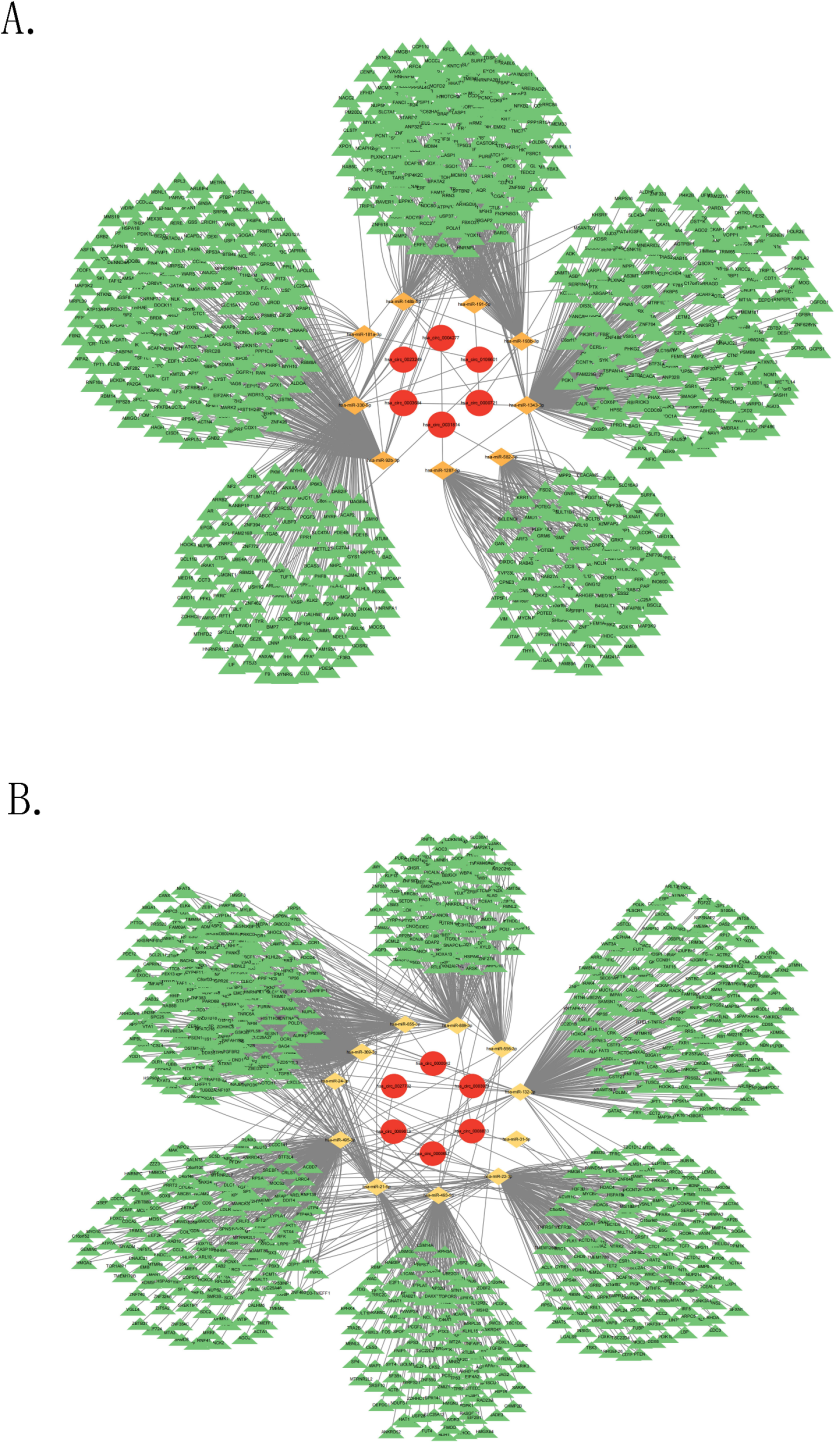
Construction of ceRNA network. **(A)** The ceRNA network based on the upregulated circRNAs, **(B)** The ceRNA network based on the downregulated circRNAs.

A ceRNA network based on the top 10 dysregulated DEcircRNAs was built to better understand the impact of the ceRNA network on the dynamic changes in gene expression regulation linked to PD in NPC patients. For the top 10 upregulated DEcircRNAs, circBank, TargetScan, CircInteractome, and RNAhybrid were applied to predict 73 overlapping miRNAs (four circRNAs without predicted miRNAs were removed). Nine miRNAs were obtained by intersecting the predicted miRNAs and DEmiRNAs. 1062 target mRNAs for these miRNAs were identified via MiRTarBase. 6 circRNAs (including hsa_circ_0004277, hsa_circ_0003684, hsa_circ_0000721, hsa_circ_0106601, hsa_circ_0023249, hsa_circ_0031814), 9 miRNAs (including hsa-miR-1287-5p, hsa-miR-1343-3p, hsa-miR-148b-5p, hsa-miR-181a-3p, hsa-miR-191-5p, hsa-miR-193b-3p, hsa-miR-330-5p, hsa-miR-582-3p, hsa-miR-92b-3p), and 1062 mRNAs were obtained (**Figure 2A**).

Regarding the top 10 downregulated DEcircRNAs, the four databases were utilized to predict 171 overlapping miRNAs (four circRNAs without predicted miRNAs were removed). 11 miRNAs were selected by intersecting the predicted miRNAs and DEmiRNAs. 1753 target mRNAs for these miRNAs were identified via MiRTarBase. 6 circRNAs (including hsa_circ_0003923, hsa_circ_0027702, hsa_circ_0000657, hsa_circ_0008833, hsa_circ_0000042, hsa_circ_0069613), 11 miRNAs (including hsa-miR-493-5p, hsa-miR-132-3p, hsa-miR-21-5p, hsa-miR-24-3p, hsa-miR-22-3p, hsa-miR-556-3p, hsa-miR-655-3p, hsa-miR-31-5p, hsa-miR-369-3p, hsa-miR-889-3p, hsa-miR-495-3p), and 1753 mRNAs were obtained (**Figure 2B**).

### mRNA GO and KEGG analyses

The potential roles of the target mRNAs were investigated via GO and KEGG analyses. KEGG analysis revealed that target mRNAs for the upregulated DEcircRNAs in the ceRNA network were enriched in oocyte meiosis, cell cycle pathways, and protein processing in the endoplasmic reticulum. GO analysis demonstrated that these target mRNAs were enriched in nuclear division, nuclear chromosome segregation, and chromosome segregation in NPC patients with PD (**Supplementary Figure 4**).

Regarding the downregulated DEcircRNAs in the ceRNA network, the KEGG analysis demonstrated that their target mRNAs were enriched in Th1 and Th2 cell differentiation, natural killer (NK) cell-mediated cytotoxicity, and axon guidance in NPC patients with PD. GO analysis indicated that these target mRNAs were enriched in antigen receptor-mediated signaling pathways and the signaling pathways of immune response-regulating and response-activating cell surface receptors (**Supplementary Figure 4**).

### RT-qPCR validation

**Figure 3:**
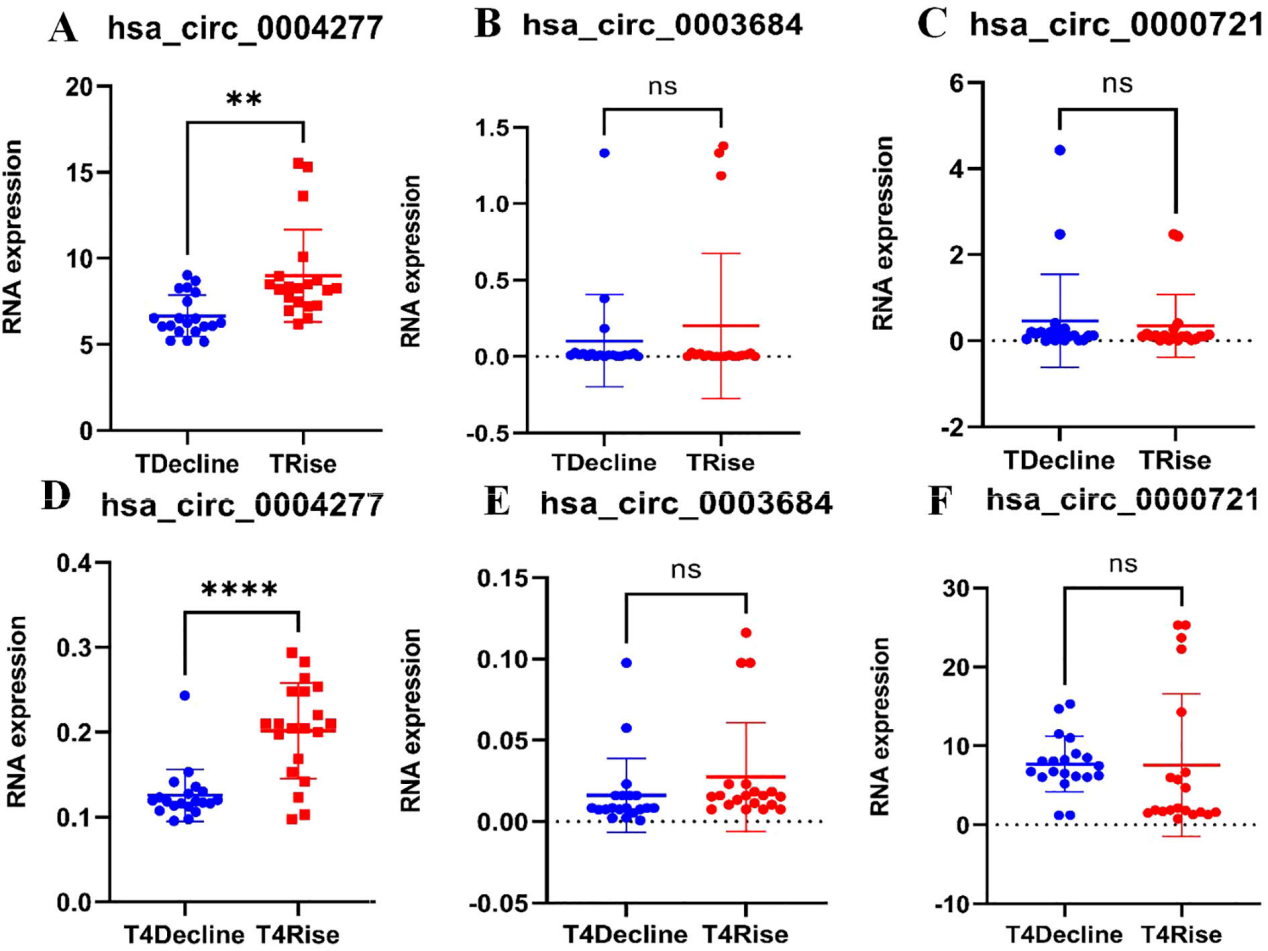
RT-qPCR validation. **(A)** Validation of hsa_circ_0004277 expression at T1 between the two classes (*P*<0.01), **(B)** Validation of hsa_circ_0003684 expression at T1 between the two classes (*P*>0.05), **(C)** Validation of hsa_circ_0000721 expression at T1 between the two classes *(P*>0.05), **(D)** Validation of hsa_circ_0004277 expression at T4 between the two classes (*P*<0.05), **(E)** Validation of hsa_circ_0003684 expression at T4 between the two classes (*P*>0.05), **(F)** Validation of hsa_circ_0000721 expression at T4 between the two classes (*P*>0.05). ^ns^P>0.05, **P<0.01, ****P<0.0001.

Blood samples at T1 and T4 from 20 Class 1 patients and 20 Class 4 patients were randomly selected (The T1 and T4 samples from the six patients previously sent for sequencing were both included). The random three upregulated circRNAs (hsa_circ_0004277, hsa_circ_0003684, and hsa_circ_0000721) identified in the ceRNA network were validated via RT-qPCR. The results of the RT-qPCR aligned with the WTS data. No significant differences in the expression levels of hsa_circ_0003684 and hsa_circ_0000721 between Class 1 and Class 4 patients at T1 and T4 were found (P > 0.05) (**Figures 3B, C, E, F**). However, as shown in **Figures 3A, D**, significant differences in the expression levels of hsa_circ_0004277 between Class 1 patients and Class 4 patients at T1 and T4 were observed (P < 0.01). The results all showed that the expression level of hsa_circ_0004277 in the ascending group was higher than that in the descending group. Among them, the expression level of the T1 rising group was 1.305 times that of the falling group, and the expression level of the T4 rising group was 1.605 times that of the falling group.

### Construction of the PPI networks

Hub genes in the ceRNA network were selected via PPI networks (**Supplementary Figure 5**). Hub genes including RPS27A, H3C13, TP53, UBA52, H4C6, BRCA1, CDK1, EZH2, PLK1, and CCNA2 were found to play vital roles in the ceRNA network based on the upregulated circRNAs. Additionally, hub genes including TP53, MYC, CTNNB1, AKT1, EGFR, EP300, RPS27A, HDAC1, ACTB, and SRC were identified in the ceRNA network based on the downregulated circRNAs.

### Construction of circRNA–RBP networks

Through a synchronous prediction, 35 RBPs were selected from the upregulated DEcircRNAs via the circAtlas and RBPsuite databases. 19 RBPs were identified from the downregulated circRNAs. The circRNA–RBP networks are visualized in **Supplementary Figure 6**.

## Discussion

### PD trajectories of NPC patients receiving radiotherapy

Most included NPC patients received 30 to 33 sessions of radiotherapy over a period of 45 to 60 days. Diagnosis was typically confirmed within three to five days of admission, with radiotherapy starting around day seven to ten and concluding by day 60 to 90. During this period, our research team was able to closely monitor changes in PD among NPC patients undergoing radiotherapy. This study identified four PD trajectories of NPC patients undergoing radiotherapy via LCGM: the decline distress group (11.0%), Class 1; the low-stable distress group (58.0%), Class 2; the no distress group (10.7%), Class 3; and the rise distress group (20.3%), Class 4. Patients with PD were mainly from Classes 1, 2, and 4, indicating the lasting PD during NPC radiotherapy. This finding is aligned with other longitudinal studies in cancer patients (15). Notably, Class 1 patients experienced high PD levels before receiving radiotherapy, which then declined steadily through the course of treatment. We hypothesized that these patients may have utilized more active coping strategies and received greater social support to help them cope with the physical and emotional changes caused by cancer. Our results indicated that compared to Class 4 and Class2 patients, those in Class 1 had higher scores for coping strategies and PSS, suggesting that adaptive coping may prevent the deterioration of psychological health (29).

### Influencing factors in PD trajectories of NPC patients receiving radiotherapy

#### Household monthly income

Based on the univariate and multivariate logistic regression analyses, patients with lower household monthly PCI tended to be defined as Class 2 or Class 4 in comparison to Class 1, thereby leading to an increased likelihood of experiencing PD. Cancer patients often encounter financial toxicity due to expensive treatment costs, thereby causing increased PD levels over time (T2–T4) and poorer psychosocial outcomes (30). Despite the comprehensive health insurance systems, the economic burden of cancer remains substantial, particularly for individuals who are unemployed or have limited income. Therefore, it is suggested that healthcare providers should guide patients seeking more financial support, such as from social platforms and charitable organizations, to help alleviate PD.

#### Coping strategies and social support

The univariate analysis demonstrated that different coping strategies and PSS were significantly linked to the PD trajectories of NPC patients undergoing radiotherapy. Logistic regression further demonstrated that compared to Class 4 and Class2, the score of confrontation coping strategy and PSSS were significant predictors of PD in Class 1. Our results demonstrated that although Class 1 patients initially exhibited high levels of PD at T1, their PD levels rapidly declined from T2 to T4. It suggested that it was potentially due to their active confrontation coping strategy. The opposite results observed in Class 4 patients may due to economic pressure and lack of social support. Our findings also indicated that patients who adopted coping strategies can be assigned to Class 2 (the stable group) possibly because they had accepted their diagnosis and were willing to follow medical advice. This may be related to their acceptance of the reality of their condition and their proactive attitude in following medical advice. This finding is consistent with previous studies (31). Maladaptive coping strategies (such as surrender and avoidance) are linked to prolonged distress, while confrontation coping strategy contributes to better patient outcomes. Other studies (32) have emphasized the crucial role of social support in mitigating PD by fostering adaptive coping strategies. Hence, clinicians need to encourage NPC patients undergoing radiotherapy to tap into their available social support networks to reduce the risk of experiencing PD.

#### Genetic factors

Numerous studies have demonstrated that circRNAs with abundant miRNA binding sites can be involved in the development and progression of various diseases through the post-transcriptional regulation of gene expression (33). However, the specific role of circRNAs in modulating PD in NPC patients receiving radiotherapy remains unestablished. The impact of genetic factors on PD in NPC patients was investigated in this study. The expression profiles of miRNA and circRNA in the rise and decline distress groups were identified via WTS, and the expression of circRNAs was further validated. Furthermore, the ceRNA and circRNA–RBP networks based on the peripheral blood samples obtained from NPC patients undergoing radiotherapy were then constructed. This study first comprehensively explored the differential expression profiles of circRNA and miRNA between the rise and decline distress groups. 600 DEcircRNAs and 123 DEmiRNA were identified from the two groups, suggesting that these genes may serve as candidate risk or protective factors for PD in these NPC patients. Additionally, significant differences in the expression level of hsa_circ_0004277 between the rise and decline distress groups were found.

A ceRNA network based on the top 10 upregulated and downregulated DEcircRNAs from the rise and decline distress groups was built in this study. Most of the circRNAs in the ceRNA network have not been reported previously. However, the reported hsa_circ_0004277 is linked to the development and progression of other cancers (34) Samples obtained from both groups at T1 and T4 were validated via RT-qPCR and statistically significant differences were found. DEmiRNAs in the ceRNA network are linked to poor prognosis in other cancers. Hsa-miR-24-3p has been shown to enhance sensitivity to radiotherapy in NPC patients (35). High expression of miR-92b-3p is linked to better prognosis in NPC patients (36). Elevated expression of hsa-miR-21-5p promotes the growth of NPC cells (37). However, previous studies have not unraveled the correlations between these factors and PD and other adverse emotions observed in NPC patients undergoing radiotherapy. Regarding the hub genes identified in the PPI network, numerous studies have demonstrated that mutations in breast cancer susceptibility gene-1 can lead to PD, anxiety, and depression in cancer patients (38). Therefore, in this study, it is suggested that the core ceRNA network, specifically the hsa_circ_0004277/hsa-miR-24-3p/BRCA1 and hsa_circ_0004277/hsa-miR-92b-3p/BRCA1 axes, is most likely to influence the progression or suppressing of the pathophysiological processes of PD in NPC patients undergoing radiotherapy.

Since RBPs have high specificity and affinity, growing attention is being paid to them. RBPs contain various proteins that can interact with transcripts at multiple levels during such RNA-driven processes as translational efficiency, mRNA localization, alternative splicing, and mRNA stability (39). In our study, the top 10 dysregulated DEcircRNAs were used to build a circRNA–RBP network. IGF2BP1 (40) and IGF2BP2 (41) were found to impact the prognosis and negative emotional outcomes in NPC patients. All human IGF2BPs have been determined as oncofetal proteins (42), and IGF2BP2, which can promote carcinogenesis by modulating cancer metabolism, is a distinct member of the IGF2BP family (43). Studies have revealed that overexpression of IGF2BP1 and IGF2BP2 increases neurogenic potentials and inhibits the astrocytic differentiation of late neural precursor cells (44). It is consistent with the functional and/or structural changes in key brain regions (thalamus, hippocampus, and astrocytes) when cancer patients experience negative emotions including anxiety, depression, and distress (45). Therefore, it is suggested that IGF2BP1 and IGF2BP2 may be involved in PD in NPC patients receiving radiotherapy.

KEGG and GO analyses unraveled that cell cycle, NK cell-mediated cytotoxicity, chromosome segregation, and antigen receptor-mediated signaling pathways were the most prominent. This finding is aligned with pathways reported in NPC patients undergoing radiotherapy in other studies. Cell cycle, one of the pathways of programmed cell death, has emerged as a promising therapeutic target. In B-cell malignancies, overexpression of anti-apoptotic proteins is linked to treatment resistance, leading to reduced radiotherapy sensitivity in NPC patients (46). Additionally, the concept of NK cell-mediated cytotoxicity has been validated by other studies, providing new insights for NK-NPC studies. NK cells greatly impact the innate immune response against tumors by recognizing and destroying tumor cells through receptor-ligand interactions (47). However, tumor cells that bind to inhibitory receptors on NK cells can send negative signals to mediate the functional suppression or exhaustion of NK cells. Consequently, during radiotherapy, the ligands of NPC tumor cells bind to inhibitory receptors on NK cells, including TIM-3, LAG3, and TIGIT, thereby reducing NK cell cytotoxicity. NK cell exhaustion leads to a loss of anti-tumor activity and negative emotions among patients (48). Chromosome segregation proteins can coordinate multiple biological processes, including apoptosis, cell proliferation, chromosomal alignment, and aggregation. Luo et al. (49) has unraveled that chromosomal expression has a protective effect on NPC by eliminating the inhibition of NPC tumors and promoting apoptosis. Moreover, some studies have suggested that antigen receptor-mediated pathways can induce apoptosis in EBV-positive NPC cells, thereby improving patient outcomes (50).

### Limitations

Homeostasis and normal physiological activities in the body are maintained by accurately regulating gene expression. In addition to investigating the sociodemographic data of PD in NPC patients undergoing radiotherapy, this study explored the influence of genetic factors. The ceRNA and circRNA-RBP networks were built to provide insights into the pathophysiology of PD in NPC patients undergoing radiotherapy. KEGG and GO analyses also provided perspectives on the mechanism and regulation of PD in these patients. However, there are several limitations in this multicenter study. First, this study involves a limited sample size, which can lead to selection bias and affect the accuracy of our conclusions. Moreover, the follow-up period is limited to 90 days at only four time points. Future studies involving larger cohorts and more extensive and frequent follow-ups are required to validate our findings. Additionally, some tools used to measure PD are self-reported, which may cause response bias. Finally, the number of blood samples used for genetic analysis is limited, and only upregulated circRNAs were validated via PCR, thereby leading to insufficiently robust evidence. The study does also not address how radiotherapy dosage or concurrent chemotherapy regimens might interact with psychological distress trajectories. Therefore, future research requires more rational design with larger samples.

## Conclusion

This study unraveled that NPC patients undergoing radiotherapy exhibited heterogeneous trajectories of PD. Therefore, screening and timely interventions in this population are required. Moreover, the expression patterns of ceRNA, and circRNA–RBP networks and pathways related to these networks indicated the potential role of circRNAs in developing PD in NPC patients during radiotherapy. This finding suggests the significant impact of genetic factors on developing PD in these patients.

## Data Availability

The original contributions presented in the study are included in the article/**Supplementary Material**. Further inquiries can be directed to the corresponding authors. The raw sequencing data involved have been uploaded to the GEO database (https://www.ncbi.nlm.nih.gov/geo/query/acc.cgi?acc=GSE303758).

## Supplementary material

Supplementary Figure 1 Expression profiles of circRNA. **(A)** Volcano plot (circRNAs), **(B)** Hierarchical clustering (circRNAs).

Supplementary Figure 2 Expression Profiles of miRNA. **(A)** Volcano plot (miRNAs), **(B)** Hierarchical clustering (miRNAs).

Supplementary Figure 3 Analysis of the maternal gene of circRNA. **(A)** Top 10 KEGG pathways of the upregulated maternal genes of DEcircRNAs, **(B)** Top 10 enriched GO terms of upregulated maternal genes of DEcircRNAs, **(C)** Top 10 KEGG pathways of the downregulated maternal genes of DEcircRNAs, **(D)** Top 10 enriched GO terms of downregulated maternal genes of DEcircRNAs.

Supplementary Figure 4 GO and KEGG analyses. **(A)** Top 10 pathways related to the upregulated DEcircRNAs in the ceRNA network, **(B)** Top 10 enriched GO terms of the upregulated DEcircRNAs in the ceRNA network, **(C)** Top 10 pathways related to the downregulated DEcircRNAs in ceRNA network, **(D)** Top 10 enriched GO terms of the downregulated DEcircRNAs in the ceRNA network.

Supplementary Figure 5 Construction of PPI networks. **(A)** The PPI network of the upregulated DEcircRNAs, **(B)** Identified top 10 hub genes by MCC and degree scores, **(C)** The PPI network of the downregulated DEcircRNAs; **(D)** Identified top 10 hub genes by MCC and degree scores.

Supplementary Figure 6 Construction of the circRNA–RBP networks. **(A)** The circRNA–RBP network based on the upregulated DEcircRNAs, **(B)** The circRNA–RBP network based on the downregulated DEcircRNAs.
