# Supplementary figures and images for: Trajectories and influencing factors of psychological distress in nasopharyngeal carcinoma patients receiving radiotherapy (incorporating genetic factors): a multicenter longitudinal study
Source: Front Oncol. 2025 Aug 27;15:1640266. doi: 10.3389/fonc.2025.1640266 (PMC12421446; doi:10.3389/fonc.2025.1640266)

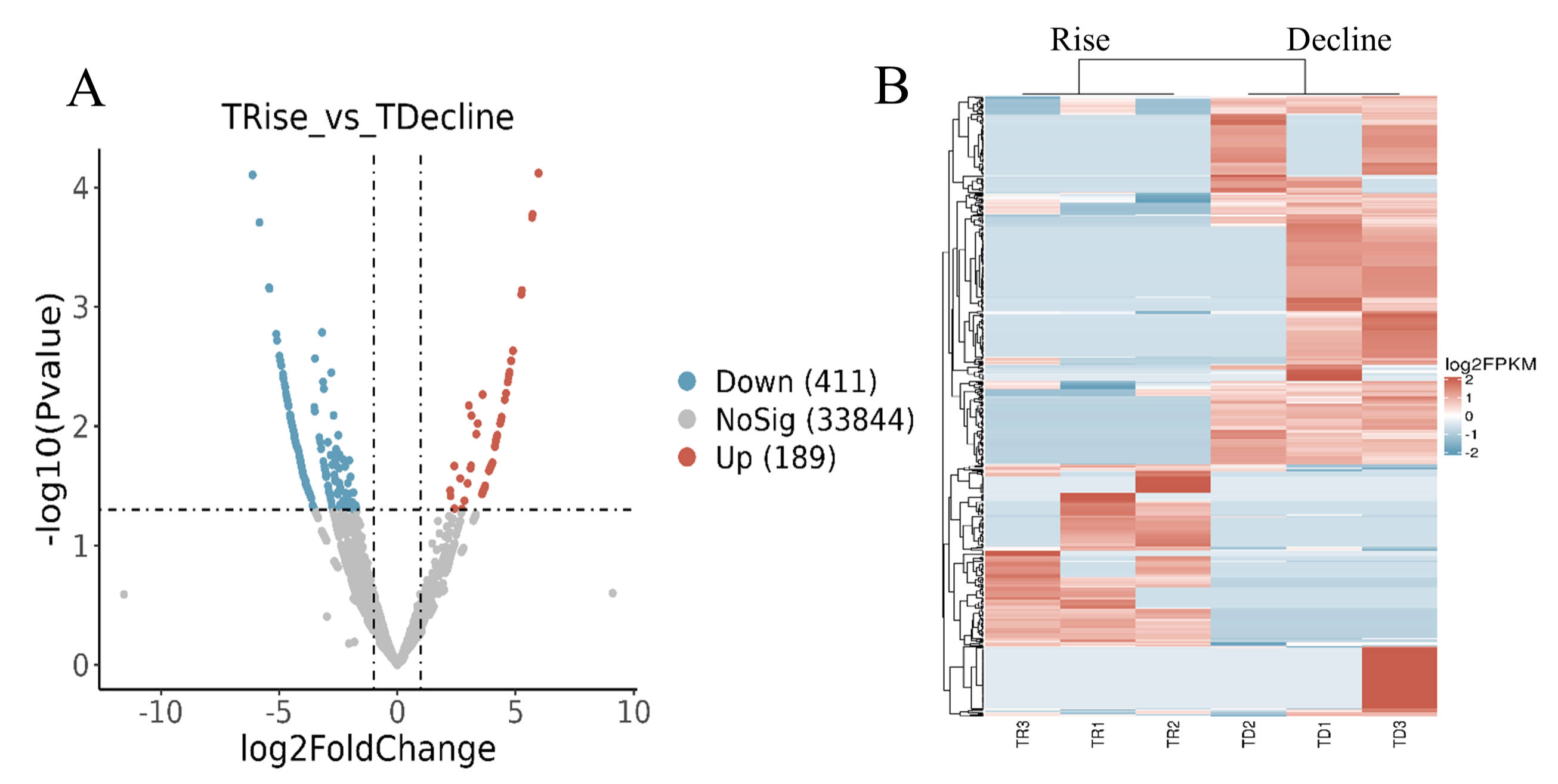

Supplement: Supplementary Figure 1 — Expression profiles of circRNA. (A) Volcano plot (circRNAs), (B) Hierarchical clustering (circRNAs). [file Image1.tif]

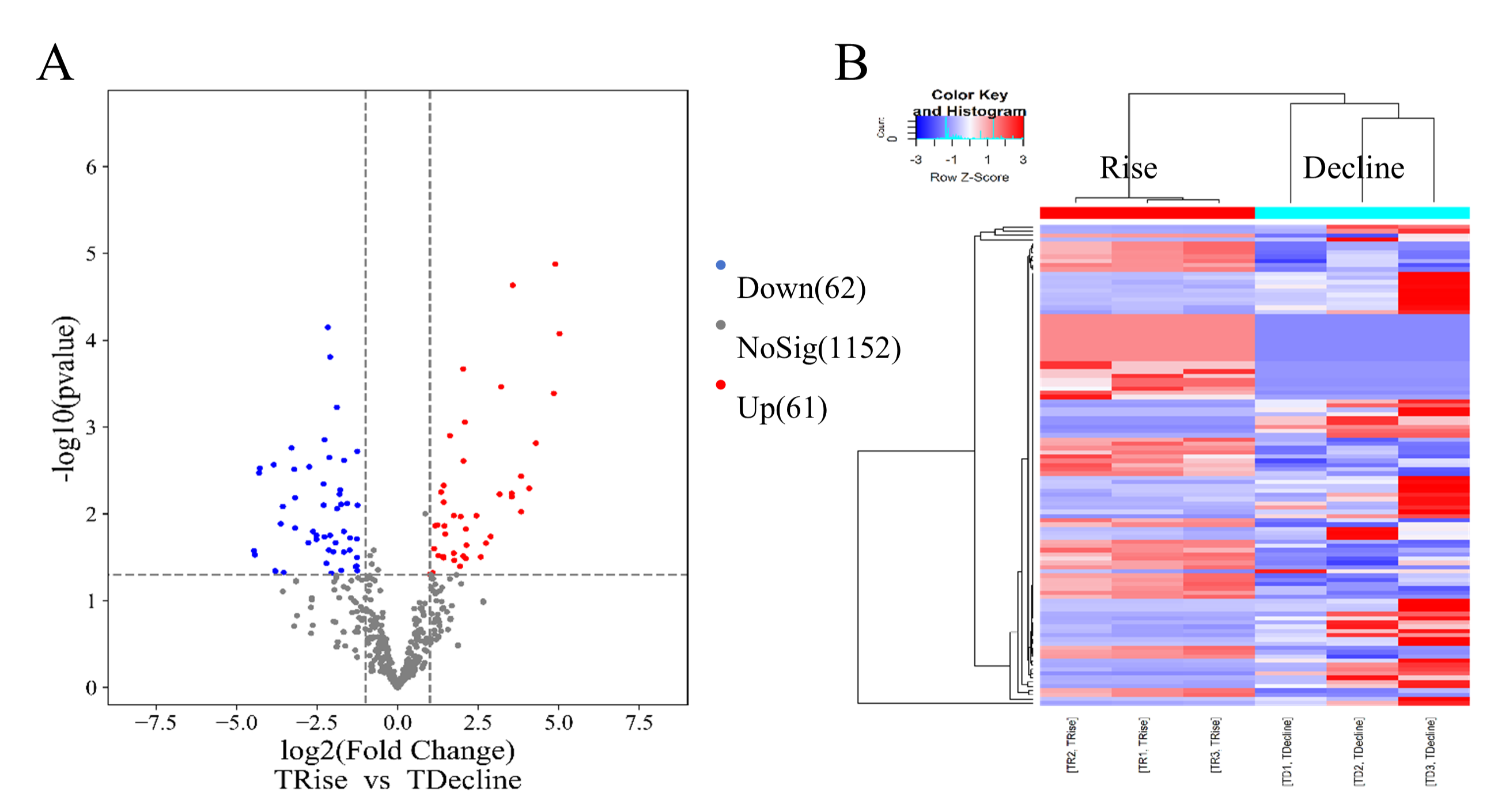

Supplement: Supplementary Figure 2 — Expression Profiles of miRNA. (A) Volcano plot (miRNAs), (B) Hierarchical clustering (miRNAs). [file Image2.tif]

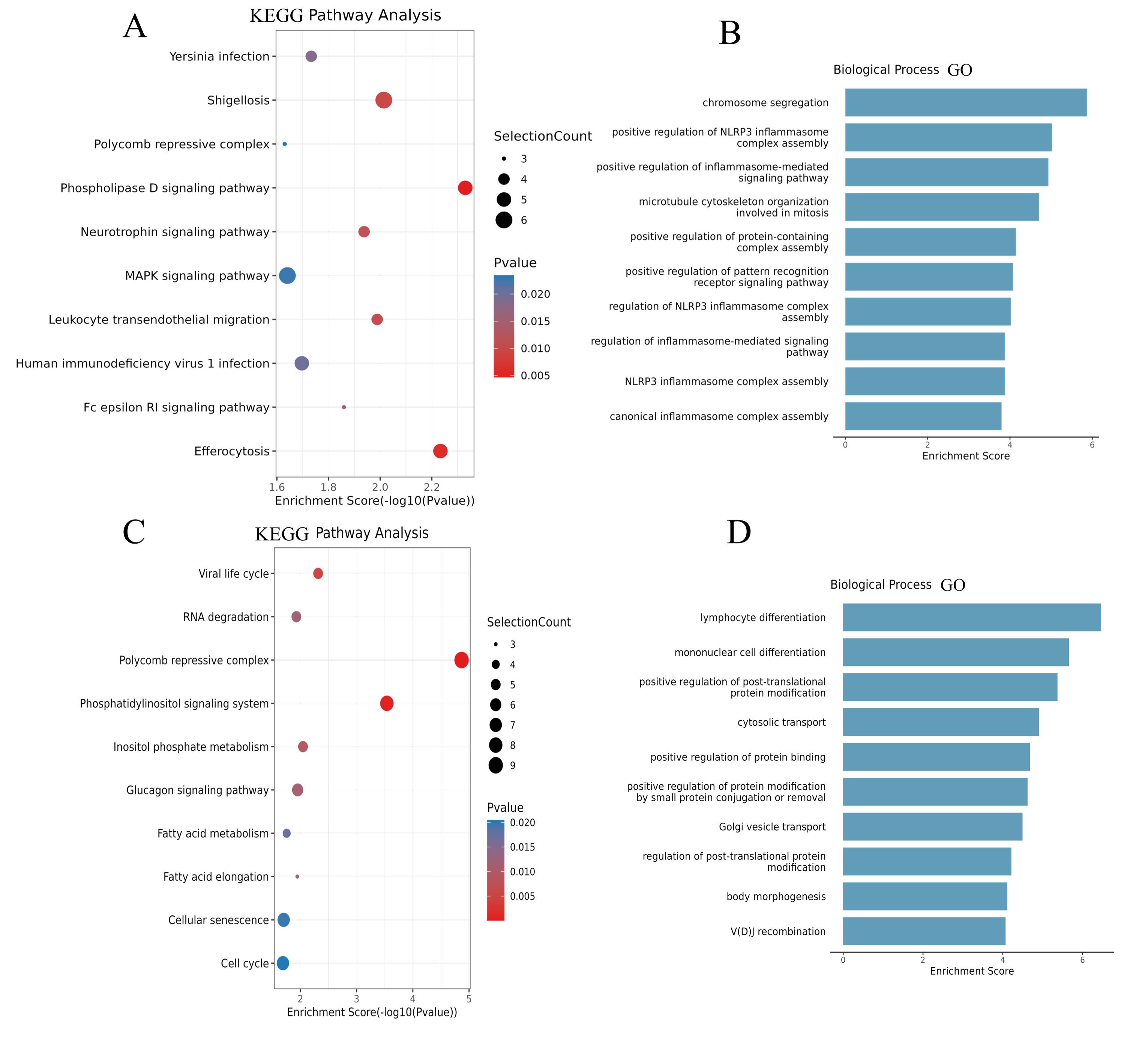

Supplement: Supplementary Figure 3 — Analysis of the maternal gene of circRNA. (A) Top 10 KEGG pathways of the upregulated maternal genes of DEcircRNAs, (B) Top 10 enriched GO terms of upregulated maternal genes of DEcircRNAs, (C) Top 10 KEGG pathways of the downregulated maternal genes of DEcircRNAs, (D) Top 10 enriched GO terms of downregulated maternal genes of DEcircRNAs. [file Image3.tif]

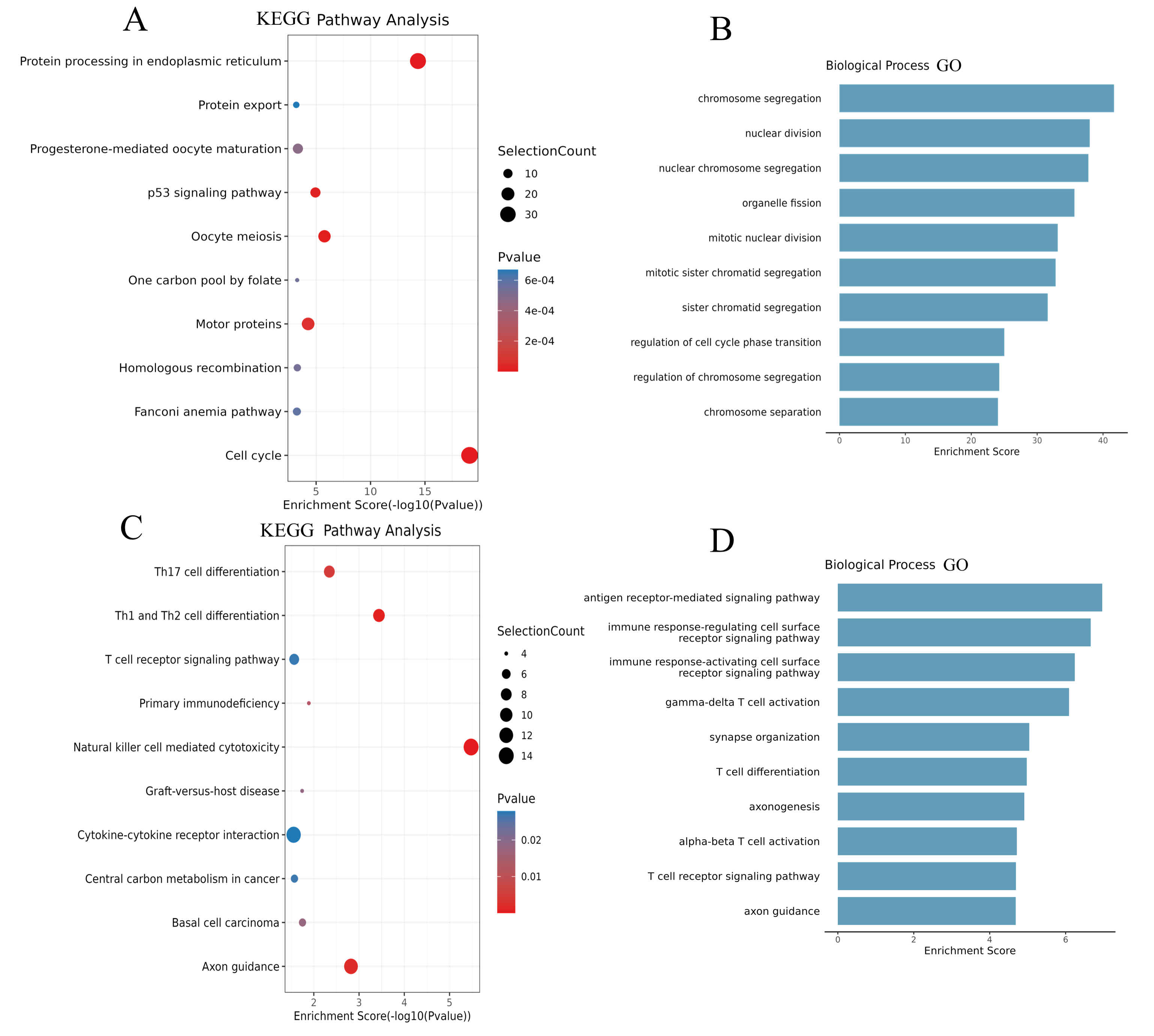

Supplement: Supplementary Figure 4 — GO and KEGG analyses. (A) Top 10 pathways related to the upregulated DEcircRNAs in the ceRNA network, (B) Top 10 enriched GO terms of the upregulated DEcircRNAs in the ceRNA network, (C) Top 10 pathways related to the downregulated DEcircRNAs in ceRNA network, (D) Top 10 enriched GO terms of the downregulated DEcircRNAs in the ceRNA network. [file Image4.tif]

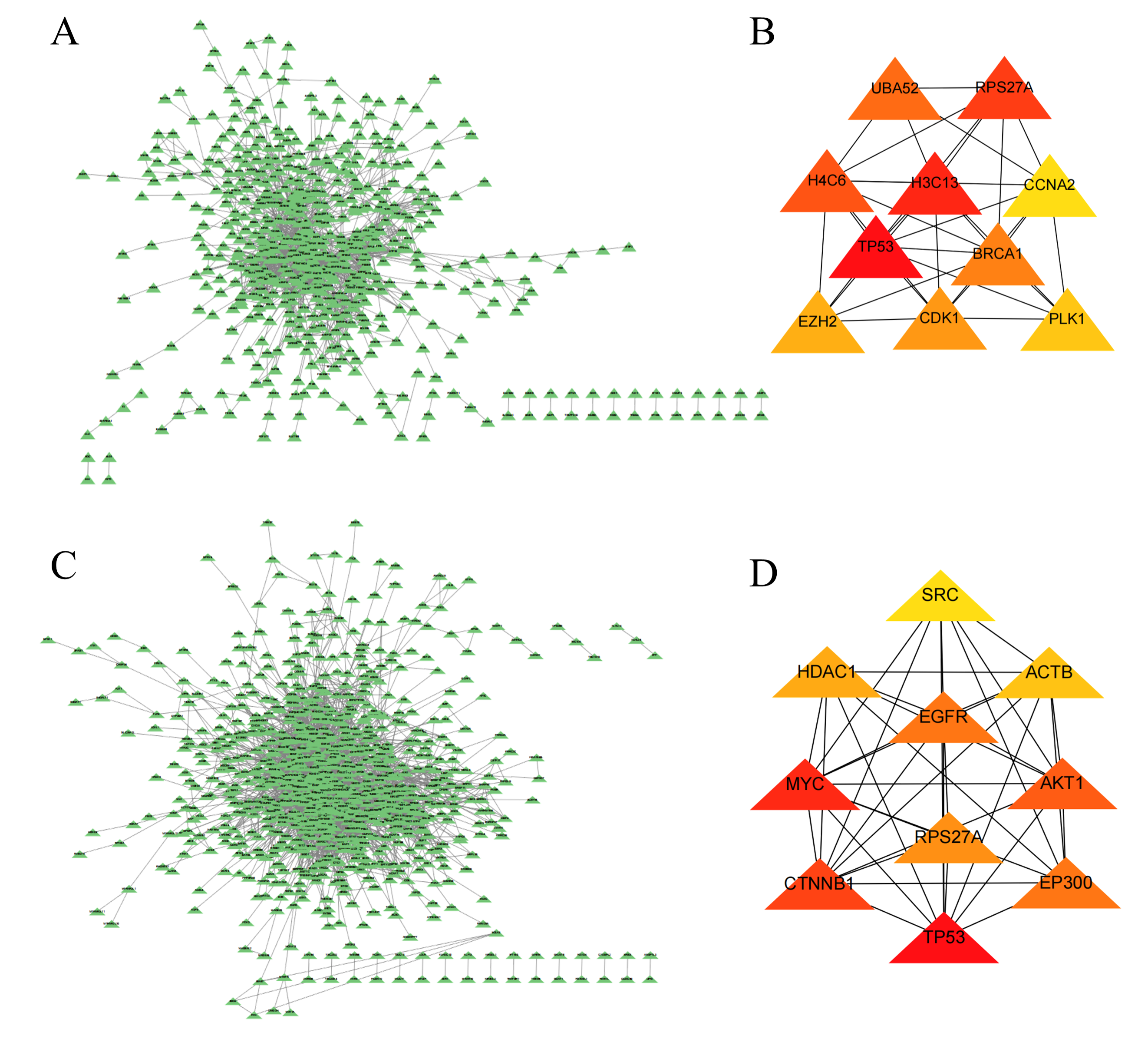

Supplement: Supplementary Figure 5 — Construction of PPI networks. (A) The PPI network of the upregulated DEcircRNAs, (B) Identified top 10 hub genes by MCC and degree scores, (C) The PPI network of the downregulated DEcircRNAs; (D) Identified top 10 hub genes by MCC and degree scores. [file Image5.tif]

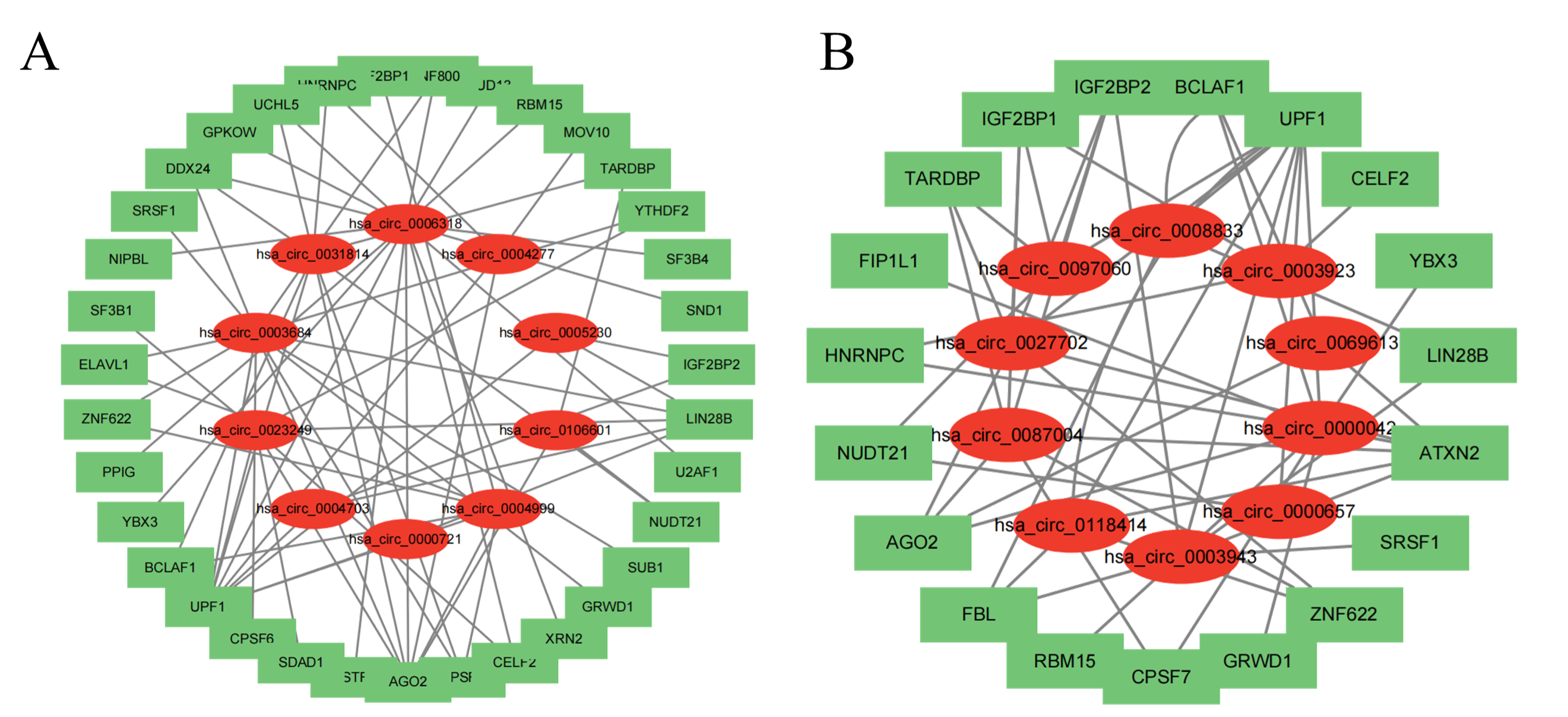

Supplement: Supplementary Figure 6 — Construction of the circRNA–RBP networks. (A) The circRNA–RBP network based on the upregulated DEcircRNAs, (B) The circRNA–RBP network based on the downregulated DEcircRNAs. [file Image6.tif]
