# Supplementary material for: Trajectories and influencing factors of psychological distress in nasopharyngeal carcinoma patients receiving radiotherapy (incorporating genetic factors): a multicenter longitudinal study
Source: Front Oncol. 2025 Aug 27;15:1640266. doi: 10.3389/fonc.2025.1640266 (PMC12421446; doi:10.3389/fonc.2025.1640266)
Supplement: Supplementary file 7 [file Table1.docx]

**Table A1. Top 10 dysregulated circRNAs**

| circRNA_ID | log2FC | Fold_Change | *P.adj value* | q_value | Up/down |
| --- | --- | --- | --- | --- | --- |
| hsa_circ_0003684 | 4.7303 | 26.5441 | 0.0037 | 0.0935 | Up |
| [hsa_circ_0005230](http://circbase.org/cgi-bin/singlerecord.cgi?id=hsa_circ_0005230) | 4.4127 | 21.2989 | 0.0084 | 0.1404 | Up |
| [hsa_circ_0023249](http://circbase.org/cgi-bin/singlerecord.cgi?id=hsa_circ_0023249) | 4.3505 | 20.4004 | 0.0095 | 0.1571 | Up |
| [hsa_circ_0004277](http://circbase.org/cgi-bin/singlerecord.cgi?id=hsa_circ_0004277) | 4.2139 | 18.5576 | 0.0121 | 0.2374 | Up |
| [hsa_circ_0004703](http://circbase.org/cgi-bin/singlerecord.cgi?id=hsa_circ_0004703) | 4.1901 | 18.2537 | 0.0126 | 0.2380 | Up |
| [hsa_circ_0000721](http://circbase.org/cgi-bin/singlerecord.cgi?id=hsa_circ_0000721) | 4.1443 | 17.6835 | 0.0142 | 0.2512 | Up |
| [hsa_circ_0106601](http://circbase.org/cgi-bin/singlerecord.cgi?id=hsa_circ_0106601) | 4.1443 | 17.6835 | 0.0142 | 0.2512 | Up |
| [hsa_circ_0031814](http://circbase.org/cgi-bin/singlerecord.cgi?id=hsa_circ_0031814) | 4.0040 | 16.0448 | 0.0206 | 0.3698 | Up |
| [hsa_circ_0006318](http://circbase.org/cgi-bin/singlerecord.cgi?id=hsa_circ_0006318) | 3.9934 | 15.9275 | 0.0208 | 0.3704 | Up |
| [hsa_circ_0004999](http://www.circbase.org/cgi-bin/singlerecord.cgi?id=hsa_circ_0004999) | 3.9653 | 15.6197 | 0.0215 | 0.3820 | Up |
| [hsa_circ_0003923](http://www.circbase.org/cgi-bin/singlerecord.cgi?id=hsa_circ_0003923) | -6.1203 | 0.0143 | 0.0001 | 0.0103 | Down |
| [hsa_circ_0118414](http://www.circbase.org/cgi-bin/singlerecord.cgi?id=hsa_circ_0118414) | 0.0316 | -4.9845 | 0.0026 | 0.0857 | Down |
| [hsa_circ_0097060](http://www.circbase.org/cgi-bin/singlerecord.cgi?id=hsa_circ_0097060) | 0.0328 | -4.9313 | 0.0029 | 0.0899 | Down |
| [hsa_circ_0003943](http://www.circbase.org/cgi-bin/singlerecord.cgi?id=hsa_circ_0003943) | 0.0373 | -4.7427 | 0.0047 | 0.1030 | Down |
| [hsa_circ_0027702](http://www.circbase.org/cgi-bin/singlerecord.cgi?id=hsa_circ_0027702) | 0.0393 | -4.6670 | 0.0058 | 0.1105 | Down |
| [hsa_circ_0000657](http://www.circbase.org/cgi-bin/singlerecord.cgi?id=hsa_circ_0000657) | 0.0408 | -4.6157 | 0.0066 | 0.1198 | Down |
| [hsa_circ_0008833](http://www.circbase.org/cgi-bin/singlerecord.cgi?id=hsa_circ_0008833) | 0.1544 | -2.6952 | 0.0081 | 0.1388 | Down |
| [hsa_circ_0087004](http://www.circbase.org/cgi-bin/singlerecord.cgi?id=hsa_circ_0087004) | 0.0423 | -4.5298 | 0.0083 | 0.1401 | Down |
| [hsa_circ_0000042](http://www.circbase.org/cgi-bin/singlerecord.cgi?id=hsa_circ_0000042) | 0.0449 | -4.4780 | 0.0092 | 0.1497 | Down |
| [hsa_circ_0069613](http://www.circbase.org/cgi-bin/singlerecord.cgi?id=hsa_circ_0069613) | 0.0459 | -4.4441 | 0.0099 | 0.1590 | Down |
