# Supplementary material for: Trajectories and influencing factors of psychological distress in nasopharyngeal carcinoma patients receiving radiotherapy (incorporating genetic factors): a multicenter longitudinal study
Source: Front Oncol. 2025 Aug 27;15:1640266. doi: 10.3389/fonc.2025.1640266 (PMC12421446; doi:10.3389/fonc.2025.1640266)
Supplement: Supplementary file 8 [file Table2.docx]

**Table A2. Primer sequence of circRNAs validated via RT-qPCR**

| circRNA | Sequence |
| --- | --- |
| hsa_circ_0004277-F | TCCACCAGCAAGTACGTAGAG |
| hsa_circ_0004277-R | GCCCTTACTCAGCTCTGCTC |
| hsa_circ_0003684-F | CGTGAGACTGCTCAGTGAGAT |
| hsa_circ_0003684-R | GTCCCCCAGGTACTTCATCAG |
| hsa_circ_0000721-F | CCTGTCCTTCTGTGGACTGC |
| hsa_circ_0000721-R | TGATCTTGGTAGTCCGCGTC |
| hsa_circ_0106601-F | AGCTGTTCTTCATCCTACAGGG |
| hsa_circ_0106601-R | TCACTGACCAGAGACATAATCAGT |
| hsa_circ_0023249-F | GCTGATGACGGCTATGGTGT |
| hsa_circ_0023249-R | TCTGTTCCACCGTCTCAGGG |
| hsa_circ_0031814-F | ACCAGTTGAAGACATACTGACG |
| hsa_circ_0031814-R | AGCTGGTCCATAGGCTTGTC |
| GAPDH-F | CCGGGAAACTGTGGCGTGATGG |
| GAPDH-R | AGGTGGAGGAGTGGGTGTCGCTGTT |
